# Switching to Faricimab in Patients with Chronic Diabetic Macular Edema Refractory to Previous Anti-VEGF Therapy: Morphological and Functional Outcomes Including Optical Coherence Tomography Angiography Parameters

**DOI:** 10.3390/jcm15218266

**Published:** 2026-10-23

**Authors:** Young-Hoon Park, Joon Young Moon, Young Gun Park

**Affiliations:** Department of Ophthalmology and Visual Science, Seoul St. Mary’s Hospital, College of Medicine, The Catholic University of Korea, 222 Banpo-daero, Seochu-gu, Seoul 06591, Republic of Korea

**Keywords:** diabetic macular edema, faricimab, angiopoietin-2, optical coherence tomography angiography, choriocapillaris

## Abstract

**Background/Objectives:** Choriocapillaris flow impairment has been implicated in the pathogenesis of diabetic macular edema (DME), and choroidal vascular insufficiency has been proposed to contribute to poor treatment responses in chronic diseases. However, whether switching to faricimab can significantly improve choroidal and retinal perfusion in eyes with chronic treatment-refractory DME is unclear. Thus, we aimed to evaluate the morphological and functional effects of faricimab in eyes with DME that showed insufficient response to previous anti-vascular endothelial growth factor (VEGF) therapy. **Methods:** This single-center retrospective study included eyes with pretreated chronic DME that were switched to faricimab and received at least three consecutive injections. The main outcome measures were changes in central macular thickness (CMT), best-corrected visual acuity (BCVA), and optical coherence tomography angiography (OCTA) parameters before and after switching to faricimab. **Results:** Thirty-eight eyes previously treated with aflibercept-insufficient responders who switched to faricimab were analyzed. The mean CMT decreased significantly from 424.9 ± 164.4 µm before switching to 330.1 ± 75.2 µm after switching to faricimab (*p* < 0.001). BCVA improved significantly from 0.63 to 0.55 logMAR (*p* = 0.027). Among the OCTA parameters, choriocapillaris flow deficit and foveal circularity showed significant improvements after switching to faricimab (*p* = 0.008 and *p* = 0.048, respectively). **Conclusions:** Switching to intravitreal faricimab resulted in significant functional and anatomical improvements in eyes with refractory DME in a real-world setting. OCTA-derived microvascular markers also showed favorable changes, which may reflect a vascular-stabilizing effect.

## 1. Introduction

Diabetic macular edema (DME) is a leading cause of visual impairment in working-age adults and a substantial public health burden. As of 2025, approximately 589 million adults worldwide live with diabetes mellitus, according to the International Diabetes Federation, and DME remains one of the most common vision-threatening complications [1,2]. Chronic hyperglycemia triggers a cascade of biochemical and hemodynamic alterations in the retinal microvasculature, including the upregulation of vascular endothelial growth factor (VEGF), disruption of endothelial tight junctions, pericyte loss, and increased vascular permeability, ultimately leading to fluid accumulation within the macula. Untreated or inadequately controlled DME can cause progressive and often irreversible loss of central vision [3,4].

The introduction of intravitreal anti-VEGF therapy has shifted the management of DME away from laser photocoagulation toward pharmacological disease modification. Current options include intravitreal anti-VEGF agents such as ranibizumab, bevacizumab, and aflibercept, together with adjunctive corticosteroid injections and focal laser photocoagulation. The Diabetic Retinopathy Clinical Research Network (DRCR.net) Protocol T study demonstrated substantial anatomical and functional improvement with ranibizumab, aflibercept, and bevacizumab, establishing anti-VEGF monotherapy as the first-line treatment for center-involving DME [5,6].

Despite these advances, 30–60% of eyes show a suboptimal or waning response to repeated anti-VEGF injections, a pattern referred to as “refractory”, “recalcitrant”, or “persistent” DME [7]. These eyes typically retain persistent or recurrent macular fluid on optical coherence tomography (OCT) despite 3–6 consecutive monthly injections. This is associated with progressive photoreceptor outer segment disruption, disorganization of the retinal inner layers, and retinal pigment epithelial atrophy, eventually leading to irreversible visual impairment [8,9]. Various treatment strategies such as more frequent injections, switching agents, and corticosteroid addition have been attempted, but their efficacy remains limited, necessitating a growing demand for new treatment options.

Faricimab (Vabysmo^®^; Genentech, Inc., South San Francisco, CA, USA), a bispecific antibody that simultaneously inhibits angiopoietin-2 (Ang-2) and VEGF-A, has emerged as a novel addition to this landscape. Its efficacy in DME was established in the phase 3 YOSEMITE and RHINE trials [10]. It demonstrated visual acuity and central macular thickness (CMT) outcomes comparable to those of aflibercept 2 mg, with the additional advantage of extended treatment intervals. Approximately 80% of faricimab-treated eyes maintained dosing every 12 or 16 weeks by year 2. However, these trials predominantly enrolled treatment-naïve patients (nearly 80%), a population that considerably differs from that in real-world clinical practice [11]. Real-world evidence on the effectiveness of faricimab as a switching agent in chronic treatment-resistant DME remains limited [12,13].

Structural OCT has long served as the primary tool for characterizing DME and monitoring the treatment response. Parameters such as central subfield thickness, intraretinal fluid, subretinal fluid, and biomarkers of chronic damage such as ellipsoid zone integrity, disorganization of the retinal inner layers, and hyperreflective retinal foci correlate with visual outcomes. OCT angiography (OCTA) adds further dimensions by allowing direct, non-invasive visualization of the retinal and choroidal microvasculature. However, most OCTA studies on diabetic eye disease have focused on diabetic retinopathy progression rather than on DME itself and have largely centered on foveal avascular zone (FAZ) changes or choroidal circulation as markers of retinopathy severity [14].

Vascular changes, particularly in choroidal perfusion, may be associated with DME. The choriocapillaris supplies the outer retina, and reduced choriocapillaris flow has been proposed to precede retinopathy progression and contribute directly to DME development through VEGF upregulation [15]. This relationship may have received less attention in DME because of technical issues, as edematous, fluid-filled retinas can easily result in projection artifacts and segmentation errors on OCTA [16]. This also makes reliable choroidal measurement more difficult to obtain in the eyes, where it may matter the most.

Most studies have relied exclusively on conventional structural OCT parameters to define and quantify treatment response [17,18]. Although some OCTA studies have examined retinal and choroidal perfusion after faricimab [19], few have focused on eyes with an insufficient response to aflibercept. This gap is important because the proposed mechanistic advantage of faricimab lies in Ang-2-mediated vascular stabilization rather than on vascular regression alone. Therefore, OCTA-derived parameters such as choriocapillaris flow deficit (CCFD), FAZ area and circularity, and retinal vessel density may capture the microvascular effects of faricimab more directly than structural thickness alone [20]. Choriocapillaris flow impairment has been implicated in the pathogenesis and progression of diabetic retinopathy and DME, and choroidal vascular insufficiency has been proposed to contribute to photoreceptor dysfunction and poor treatment responses in chronic diseases [21]. However, whether switching to faricimab can significantly improve choroidal and retinal perfusion in eyes with an insufficient response to aflibercept remains undetermined.

In this study, we evaluated the morphological, functional, and microvascular outcomes after switching to faricimab in eyes with chronic DME that had responded insufficiently to prior anti-VEGF therapies in a real-world setting. By assessing OCTA-derived perfusion metrics alongside standard CMT and best-corrected visual acuity (BCVA) outcomes, we aimed to gain further mechanistic insights into dual Ang-2/VEGF-A inhibition and help guide clinical decisions when switching to faricimab in this hard-to-treat population.

## 2. Materials and Methods

### 2.1. Ethical Statement

All procedures were conducted in accordance with the Declaration of Helsinki (1964) and its later amendments. This retrospective study was conducted using data from medical records. Institutional Review Board/Ethics Committee approval was obtained from Seoul St. Mary’s Hospital, The Catholic University of Korea. The Institutional Review Board of Seoul St. Mary’s Hospital, The Catholic University of Korea, waived the need for informed consent owing to the retrospective nature of the study (KC25RASI0519).

### 2.2. Study Design and Patients

This was a retrospective, single-center study conducted at Seoul St. Mary’s Hospital, Seoul, Republic of Korea. Medical records of patients who visited the retina clinic between September 2024 and May 2025 were reviewed.

Eyes were included if they had clinically significant, center-involving DME with CMT > 300 µm on swept-source OCT. Refractory DME was defined by a persistent CMT > 300 µm with <10% reduction, in line with previously used response-based criteria [22,23], assessed 1–2 months after at least three consecutive aflibercept (Eylea, 2 mg) injections. Although monthly dosing is generally preferred, intervals of 6–8 weeks were used in routine practice to reduce treatment burden in patients with chronic conditions. Eyes meeting these criteria were switched to faricimab. To avoid inter-eye correlation, only one eye per patient was included.

Eyes were excluded if they had any of the following: (1) macular edema attributable to causes other than diabetic retinopathy, such as retinal vein occlusion, uveitis, or age-related macular degeneration; (2) significant media opacity precluding adequate OCT or OCTA image acquisition (signal strength index < 70/100) and eyes without analyzable OCTA images at either baseline or follow-up; (3) history of intraocular surgery including vitrectomy and glaucoma surgery; and (4) concurrent use of intravitreal corticosteroids or other adjunctive therapy during the observation period that could independently affect macular thickness or vascular parameters.

### 2.3. Treatment Protocol

All eyes that met the switching criteria were administered intravitreal faricimab. The baseline was defined as the time of the first faricimab injection. The eyes subsequently received three consecutive injections at 6–8-week intervals, and a follow-up examination was performed 12–16 weeks after the third injection.

### 2.4. Outcome Measures

Baseline demographic and clinical data, including age, sex, HbA1c level, number of prior anti-VEGF injections, history of panretinal photocoagulation, and diabetic retinopathy stage, were recorded for each patient. All patients underwent comprehensive ophthalmic examinations at baseline and follow-up, including BCVA measurement, slit-lamp biomicroscopy, dilated fundus examination, and spectral-domain OCT and OCTA. Imaging was performed using a swept-source OCT device (DRI OCT Triton, Topcon Co., Tokyo, Japan) and analyzed using IMAGEnet 6 (version 1.14).

### 2.5. Optical Coherence Tomography Angiography

OCTA scans were obtained with DRI OCT Triton (Topcon) using a 4.5 × 4.5-mm square centered on the fovea. OCTA images were evaluated using automatic segmentation. We analyzed the vascular density of the superficial and deep retinal vascular zones (i.e., the SCP and DCP) within a central 1-mm circle and four quadrants (superior, inferior, temporal, and nasal) within a surrounding 3-mm circle, using the built-in software program.

Perfusion density in the superficial and deep capillary plexuses was calculated automatically by the device for each sector (superior, nasal, temporal, inferior, and central). FAZ area, perimeter, and circularity were likewise obtained from the device output.

CCFD was quantified from en face OCTA images using ImageJ (version 1.54; National Institutes of Health, Bethesda, MD, USA). After manual correction of segmentation errors, images were binarized using the Phansalkar local thresholding method, and CCFD was calculated as the percentage of flow deficit area relative to the total analyzable area. Scans with significant motion artifact or uncorrectable segmentation error were excluded. Image analysis was performed by two retina specialists masked to the time point. All OCTA images were graded independently by two masked retina specialists, with discrepancies resolved by consensus or by adjudication with a third grader. Images with significant motion artifact, segmentation error, or signal strength index < 70 were excluded from analysis.

Adverse events including intraocular inflammation, endophthalmitis, elevated intraocular pressure, and any other ocular or systemic events were recorded from the medical records throughout the follow-up period.

### 2.6. Statistical Analysis

Statistical analysis was performed using SPSS version 24.0 (IBM Corp., Armonk, NY, USA). Only one eye per patient was included in the analysis to avoid inter-eye correlation. Normality was confirmed using the Shapiro–Wilk test. Changes between baseline and follow-up were compared using the paired *t*-test, and the association between OCTA parameters and final visual acuity was assessed using Pearson correlation. In some analyses, partial correlation analyses were performed with adjustment for age, HbA1c, and the number of previous injections. A *p*-value < 0.05 was considered statistically significant.

## 3. Results

### 3.1. Patient Demographics and Baseline Characteristics

A total of 38 eyes from 38 patients with refractory DME were included in the analysis. The mean patient age was 58.2 ± 10.1 years, and the mean HbA1c was 7.19% ± 1.63%. By severity, 27 eyes had severe non-proliferative diabetic retinopathy and 11 eyes had proliferative diabetic retinopathy; no eyes had mild/moderate NPDR. Eyes had received a mean of 6.05 ± 1.9 prior aflibercept injections before switching to faricimab. At baseline, the mean BCVA was 0.63 ± 0.23 logMAR, and the mean CMT was 424.9 ± 164.4 µm (Table 1).

### 3.2. Visual and Anatomical Outcomes

**Figure 1:**
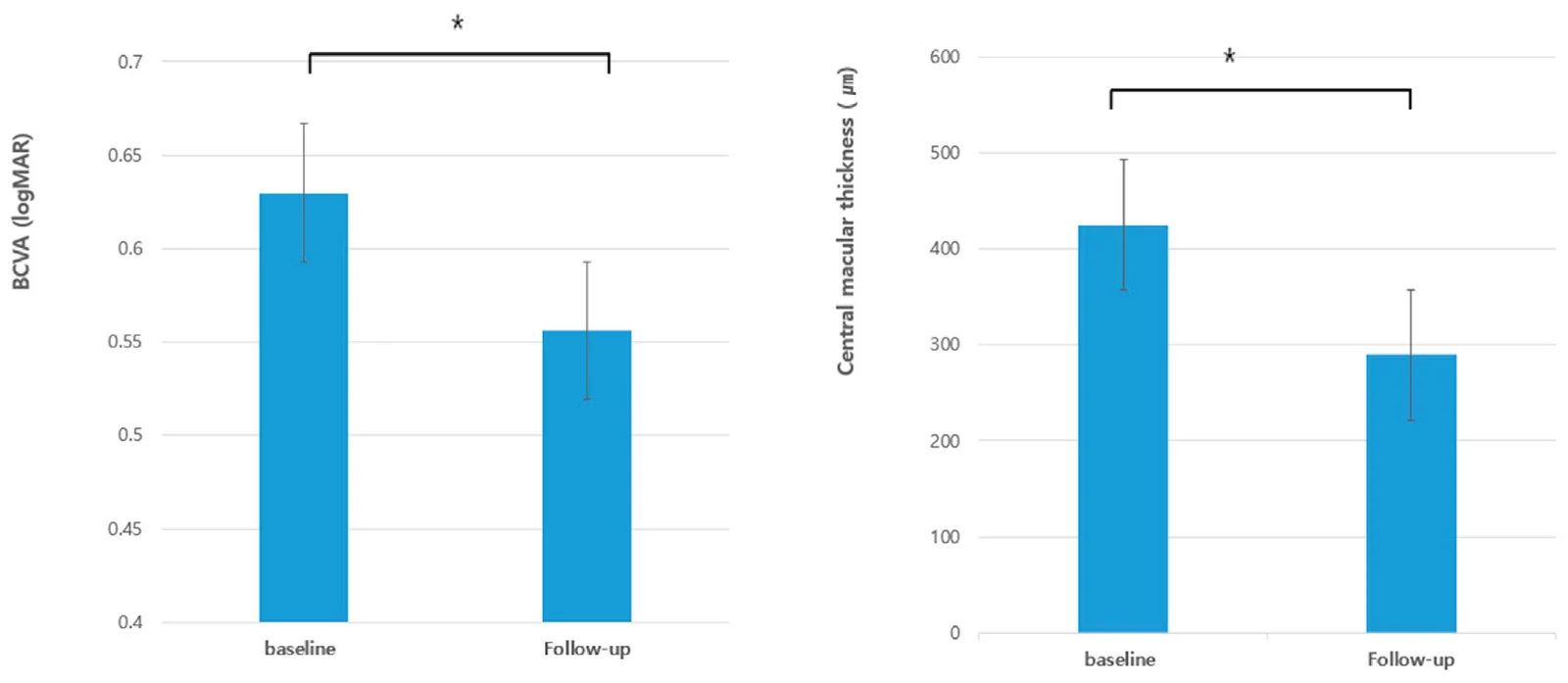
Changes in BCVA and CMT before and after switching to faricimab. Both BCVA (**left**) and CMT (**right**) showed significant improvement at follow-up compared with baseline (all * *p* < 0.05).

After three consecutive faricimab injections, BCVA improved from 0.63 ± 0.23 to 0.55 ± 0.24 logMAR, and CMT decreased from 424.9 µm to 330.1 µm. Both parameters showed statistically significant changes (*p* = 0.027 and <0.001, Figure 1).

### 3.3. OCTA Perfusion Density

The perfusion density in the superficial and deep capillary plexuses did not change significantly in any sector (center, superior, temporal, inferior, or nasal) between baseline and follow-up (all *p* > 0.05; Table 2). This pattern was consistent across both layers, indicating that the treatment effect of faricimab was not captured by conventional perfusion density metrics.

### 3.4. FAZ Morphology and Choriocapillaris Flow Deficit

Several microvascular parameters related to FAZ and choriocapillaris changed significantly, in contrast to perfusion density. The FAZ area decreased numerically from 0.48 ± 0.18 mm^2^ to 0.37 ± 0.24 mm^2^, but this change was not significant (*p* = 0.202). FAZ perimeter decreased significantly from 4.32 ± 1.63 mm to 3.27 ± 1.18 mm (*p* = 0.044), and FAZ circularity improved from 0.41 ± 0.19 to 0.45 ± 0.19 (*p* = 0.048), reflecting a more regular capillary contour around the fovea after switching. CCFD also decreased significantly, from 46.72% ± 6.44% at baseline to 39.59% ± 4.34% at follow-up (*p* = 0.008), representing the largest and most consistent vascular change observed in this study (Table 3).

### 3.5. Correlation with Final Visual Acuity

The correlations between OCTA parameters and final visual acuity are summarized in Table 4. Baseline CCFD was significantly correlated with the final visual acuity (r = 0.528, *p* = 0.029); however, this association weakened and lost significance at follow-up (r = 0.411, *p* = 0.145). FAZ circularity showed the opposite pattern; it did not correlate with visual acuity at baseline (r = 0.154, *p* = 0.555) but was significantly correlated at follow-up (r = 0.520, *p* = 0.047). The FAZ area and perimeter showed no significant correlation with visual acuity at either time point (Table 4).

### 3.6. Safety

No cases of intraocular inflammation, endophthalmitis, or faricimab-associated retinal vasculitis or occlusion were observed during the follow-up period, and no systemic adverse events were reported.

## 4. Discussion

In this retrospective study of 38 eyes with chronic DME refractory to prior anti-VEGF therapy, switching to faricimab was associated with significant improvements in BCVA and CMT, along with favorable changes in several OCTA-derived vascular parameters, most notably a reduction in CCFD and an increase in FAZ circularity. In contrast, the conventional perfusion density in the superficial and deep capillary plexuses remained largely unchanged. In contrast, perfusion density in the superficial and deep capillary plexuses remained largely unchanged. The BCVA gain (0.08 logMAR, about 4 letters) was statistically significant but clinically modest.

To minimize motion artifacts, we used eye-tracking software during acquisition and applied post-acquisition software corrections to each scan. However, some residual artifacts likely remained despite these efforts. Larger retinal cysts and intraretinal fluid can distort the segmentation of retinal layers and introduce segmentation errors [15,19]. In DME, segmentation errors most often occur at the inner or outer plexiform layer, or both, and automated segmentation tends to be less accurate in eyes with large cysts and increased CMT because the apical portion of a large cyst can distort the overlying retinal layers and disrupt the default outer plexiform layer–outer nuclear layer slab.

The changes in FAZ perimeter (*p* = 0.044) and circularity (*p* = 0.048) were borderline and should be interpreted with caution; nevertheless, the improvement in circularity without a corresponding change in the FAZ area may be worth examining. Tang et al. reported that FAZ circularity, but not FAZ area, was associated with visual acuity in diabetic eyes [24], and our results reflect the same pattern, although this correlation was also borderline (*p* = 0.047); FAZ circularity at follow-up was significantly correlated with final visual acuity, whereas FAZ area was not. Endo et al. [25] similarly identified FAZ circularity as a factor associated with visual impairment in diabetic eyes, proposing that it captures capillary loss and remodeling at the foveal border, essentially reflecting the disruption of the terminal perifoveal capillaries. In chronic DME, recurrent macular fluid accumulation may stretch and distort the terminal capillaries by reducing their elasticity and pulling the FAZ contour into an irregular shape. If faricimab promotes greater vascular stability through Ang-2 inhibition, the mechanical stress on the perifoveal capillaries would be partially relieved. In addition, it allows the FAZ border to regain a more physiological contour. This interpretation is consistent with the structural reason for the change in FAZ circularity observed in our cohort.

The reduction in CCFD was the most consistent vascular finding in this study, which aligns with prior evidence relating choroidal perfusion to DME pathogenesis. Chen et al. [26] showed that CCFD was associated with both diabetic retinopathy progression and DME development. They suggested that choroidal ischemia is an active contributor to macular edema. This is consistent with the broader concept of diabetic choroidopathy, in which the choroidal microvasculature undergoes structural remodeling as part of the systemic diabetic process rather than as a downstream consequence of retinal disease. Choriocapillaris ischemia induces outer retinal hypoxia, upregulates VEGF expression, and results in a low-grade inflammatory state. These effects promote fluid leakage into the macula. Consequently, agents targeting both VEGF-driven permeability and Ang-2-mediated vascular instability would be particularly appropriate for modulating the cycle at the choroidal level. This may explain why CCFD showed the most notable improvement among the vascular parameters measured.

We focused on the effect of faricimab on capillary architecture and choroidal flow, not on overall vessel density in patients with refractory DME who received conventional anti-VEGF. This is related to the mechanism of action of faricimab. Beyond blocking VEGF-A, Ang-2 inhibition has been proposed to stabilize an already existing vascular network rather than induce regression [21,27]. If stability rather than regression is the relevant mechanism, structural OCT can confirm fluid clearance but provides limited information regarding whether the underlying architecture has changed, which is where OCTA, through detection of FAZ morphology and choriocapillaris signals, may be more helpful.

One possible explanation is that Ang-2, independent of VEGF, destabilizes pericyte-endothelial coupling and causes pericyte loss in diabetic retinas [28]. Moreover, patients already refractory to VEGF-directed therapy have residual pathology that may be influenced more by this Ang-2-driven component (pericyte instability and low-grade inflammation) than by VEGF itself. If this is the case, FAZ circularity and perimeter may reflect this residual component.

The FAZ circularity finding fits a pattern reported elsewhere, where circularity, rather than area, tracks visual acuity most closely in diabetic eyes [25]. In our cohort, only circularity, not area, was correlated with the final BCVA. One interpretation is that mechanical changes occur because the repetitive occurrence of macular fluid stretches the terminal perifoveal capillary arcades. These arcades may partially recover once the edema resolves and vascular stability improves. The resulting shrinkage in the FAZ perimeter, along with the rise in circularity, is consistent with the selective recovery of the most distorted segments rather than with generalized capillary loss. However, this remains speculative, because we could not directly visualize capillary-level remodeling in our data.

The reduction in CCFD was the largest and most consistent vascular change observed in this study. However, projection artifacts and segmentation errors are inherently more common in eyes with persistent fluid, and part of the apparent CCFD and FAZ improvement may reflect better image quality after edema resolution rather than true microvascular change, which we cannot exclude. The CCFD change (*p* = 0.008) was the most robust of the OCTA findings, although it would not necessarily remain significant after correction across all OCTA comparisons. By contrast, perfusion density showed no significant change in either the superficial or deep capillary plexuses. Given that this metric is particularly sensitive to motion and segmentation artifacts in fluid-filled eyes, measurement noise may have obscured an effect that was real but too small to detect. Therefore, the perfusion density result should not necessarily be taken as evidence that faricimab has no effect on retinal vessel density [26].

The correlation analysis suggested two distinct patterns. Baseline CCFD was associated with the final visual acuity, an association that was weakened at follow-up. FAZ circularity showed the reverse pattern; it was unrelated to vision at baseline but significantly correlated at follow-up. This demonstrates the possibility that CCFD reflects a pre-existing choroidal compromise that restrains visual recovery, whereas FAZ circularity better reflects the response of the retina to treatment itself. However, further confirmation in larger cohorts is required.

Our anatomical and functional results are broadly consistent with other real-world reports of switching to faricimab in recalcitrant DME [12,29] and with reviews suggesting that extended dosing intervals achieved in YOSEMITE and RHINE often translate into real-world practice [30]. However, these trials predominantly enrolled treatment-naïve eyes or eyes without recent intravitreal therapy, with limited representation of eyes that had already shown resistance to multiple agents. Our cohort, with a mean of 6.05 prior aflibercept injections, represents an underrepresented group. From this perspective, the pivotal clinical trials and the present study offer complementary insights. The trials demonstrated the strength of faricimab as first-line therapy, whereas our data justify its use as a switching agent, when prior anti-VEGF treatment has generated suboptimal results. The main contribution of this study is the assessment of CCFD and FAZ morphology in a population defined by an insufficient response to aflibercept.

This study had some limitations. The cohort was small, with a short follow-up period, and the design was retrospective and single-center; therefore, none of the vascular changes observed, particularly the CCFD shift, can be considered durable. Without a comparator group, the observed changes cannot be attributed specifically to faricimab, and regression to the mean, natural fluctuation, prior treatment effects, and edema-related changes in OCTA measurements may have contributed. Given the small sample size, we did not apply corrections for multiple comparisons across the OCTA parameters tested, and several significant findings were borderline (FAZ perimeter, *p* = 0.044; circularity, *p* = 0.048). In addition, FAZ circularity at follow-up was unexpectedly positively correlated with final logMAR BCVA, meaning that higher circularity was associated with worse vision; given its borderline significance, this may be a chance finding. Therefore, OCTA results should be interpreted as hypothesis-generating rather than confirmatory. OCTA is also inherently less reliable than structural OCT in eyes with persistent fluid, owing to the higher frequency of projection and segmentation artifacts. Larger prospective studies with longer follow-up periods, prespecified correction for multiple testing, and OCTA-specific endpoints are needed to confirm whether these findings are reproducible.

Nevertheless, in practical terms, the most useful finding here may be that OCTA measures of capillary architecture and choroidal flow tracked the effect of faricimab more consistently than structural thickness or perfusion density in a population where prior anti-VEGF had already exhausted much of the structural response. Our data support faricimab as a reasonable switching option for refractory DME and suggest that FAZ circularity and CCFD may be worth evaluating as complementary endpoints in future adequately powered studies.

In summary, switching to faricimab was associated with significant improvements in BCVA, CMT, and several other vascular parameters, particularly CCFD and foveal circularity, in a cohort of eyes with refractory DME. Final visual acuity correlated with both baseline CCFD and final FAZ circularity, supporting the role of these OCTA parameters as complementary markers of treatment response and visual prognosis in this setting.

## 5. Conclusions

In this retrospective cohort of eyes with refractory DME, switching to faricimab was associated with significant functional and anatomical improvement after three loading injections. A reduction in CCFD and increase in FAZ circularity were also observed as exploratory findings. Switching to faricimab may be a reasonable option for patients with refractory DME, and larger, controlled studies are needed to confirm these findings.

## Figures and Tables

**Table 1:** Baseline characteristics.

Patient Demographics	
eyes (*n*)	38
age	58.2 ± 10.1
HbA1c	7.19 ± 1.63
best-corrected visual acuity (logMAR)	0.63 ± 0.23
central macular thickness (µm)	424.9 ± 164.4
**stage of diabetic retinopathy**	
mild/moderate NPDR	0
severe NPDR	27
PDR	11
number of previous injections	6.05 ± 1.9

logMAR, logarithm of the Minimum Angle of Resolution; NPDR, non-proliferative diabetic retinopathy; PDR, proliferative diabetic retinopathy.

**Table 2:** Changes in perfusion density.

Parameters	Baseline	Follow-Up	*p*-Value
superficial vascular layer (%)			
center	17.45 ± 4.14	17.50 ± 5.57	0.64
superior	43.89 ± 4.11	41.23 ± 4.59	0.09
temporal	43.23 ± 4.19	44.73 ± 1.92	0.11
inferior	41.49 ± 2.71	41.18 ± 5.11	0.51
nasal	41.09 ± 3.99	40.53 ± 5.35	0.72
deep vascular layer (%)			
center	16.01 ± 4.27	14.73 ± 5.44	0.68
superior	42.35 ± 7.18	41.69 ± 7.54	0.95
temporal	44.31 ± 4.11	44.93 ± 5.30	0.44
inferior	42.43 ± 3.12	41.98 ± 8.20	0.57
nasal	41.98 ± 3.28	41.27 ± 5.14	0.79

**Table 3:** Changes in FAZ parameters and choriocapillaris flow deficit.

Parameters	Baseline	Follow-Up	*p*-Value
fovea avascular zone			
area (mm^2^)	0.48 ± 0.18	0.37 ± 0.24	0.202
perimeter (mm)	4.32 ± 1.63	3.27 ± 1.18	0.044 *
circularity	0.41 ± 0.19	0.45 ± 0.19	0.048 *
choriocapillaris flow deficit (%)	46.72 ± 6.44	39.59 ± 4.34	0.008 *

* *p* < 0.05 indicates statistical significance.

**Table 4:** Correlation analysis of OCT-angiography parameters with final visual acuity.

Parameters	Baseline		Follow-Up	
fovea avascular zone	*r*	*p*-value	*r*	*p*-value
area (mm^2^)	0.440	0.077	0.372	0.172
perimeter (mm)	−0.278	0.280	−0.421	0.118
circularity	0.154	0.555	0.520	0.047 *
choriocapillaris flow deficit (%)	0.528	0.029 *	0.411	0.145

*r*, Pearson correlation coefficient; * *p* < 0.05 indicates statistical significance.

## Data Availability

The datasets analyzed during the current study are available from the corresponding author on reasonable request.

## Abbreviations

The following abbreviations are used in this manuscript:

Ang-2	angiopoietin-2
BCVA	best-corrected visual acuity
CCFD	choriocapillaris flow deficit
CMT	central macular thickness
DME	diabetic macular edema
FAZ	foveal avascular zone
OCT	optical coherence tomography
OCTA	optical coherence tomography angiography
VEGF	vascular endothelial growth factor
